# Endothelin Inhibitors in Chronic Kidney Disease: New Treatment Prospects

**DOI:** 10.3390/jcm13206056

**Published:** 2024-10-11

**Authors:** Agata Rakotoarison, Marta Kepinska, Andrzej Konieczny, Karolina Władyczak, Dariusz Janczak, Agnieszka Hałoń, Piotr Donizy, Mirosław Banasik

**Affiliations:** 1 Department of Nephrology and Transplantation Medicine, Wroclaw Medical University, Borowska 213, 50-367 Wrocław, Poland; andrzej.konieczny@umw.edu.pl; 2 Department of Pharmaceutical Biochemistry, Faculty of Pharmacy, Wroclaw Medical University, Borowska 211a, 50-556 Wroclaw, Poland; marta.kepinska@umw.edu.pl; 3 Clinical and Experimental Pathology, Wroclaw Medical University, 50-367 Wrocław, Poland; karolina.wladyczak@gmail.com (K.W.); agnieszka.halon@umw.edu.pl (A.H.); piotr.donizy@umw.edu.pl (P.D.); 4 Department of General, Vascular and Transplant Surgery, Wroclaw Medical University, 50-367 Wrocław, Poland; dariusz.janczak@umw.edu.pl

**Keywords:** endothelins, ETAR, ETBR, chronic kidney disease, Nephropathy IgA, diabetic nephropathy, FSGS, proteinuria

## Abstract

The endothelin system is reported to play a significant role in glomerular and tubulointerstitial kidney disease. In the kidney, endothelins are produced in mesangial cells and the glomerular basement membrane by the endothelium and podocytes. The endothelin system regulates glomerular function by inducing proliferation, increasing permeability and in effect proteinuria, and stimulating inflammation, tubular fibrosis, and glomerular scarring. Endothelin A receptor antagonists have been proven to delay the progression of chronic kidney disease and play a protective role in immunoglobulin A nephropathy, focal segmental glomerulosclerosis, and diabetic nephropathy. There are several ongoing research studies with ETAR antagonists in nondiabetic nephropathy, Alport disease, vasculitis and scleroderma nephropathy, which results are promising. Some reports suggest that the endothelin system might contribute to ischemia–reperfusion injury, acute graft rejection and deterioration of graft function. Endothelin inhibition in renal transplantation and its influence on graft survival is the future direction needing further research. The most frequent side effects associated with ETAR antagonists is fluid retention. Additionally, it should be considered if selective ETAR antagonists therapy needs to be co-administered with sodium-glucose co-transporter 2 inhibitors, renin–angiotensin–aldosterone inhibitors or diuretics and which patients should be recruited to such treatment to minimize the risk of adverse outcomes.

## 1. Introduction

Endothelins (ET) are a family of vasoconstrictive molecules produced mostly in the endothelium of the vascular system. It is reported that the endothelin system plays a crucial role in both glomerular and tubulointerstitial kidney disease. In the kidney ETs are produced within the mesangial cells and in the glomerular basement membrane (GBM), by the endothelium and podocytes. They act via receptors for ETsETAR and ETBR located in the epithelial cells, tubules, and collecting ducts. ETsregulate glomerular function by inducing proliferation, increasing membrane permeability and in effect proteinuria, and stimulating inflammation, tubular fibrosis, and glomerular scarring [1,2]. Several studies show that ETAR inhibition may have a positive impact on both the preservation of glomerular function and proteinuria reduction. ETAR antagonists have been proven to delay the progression of chronic kidney disease (CKD) and play a protective role in IgA nephropathy (IgAN), focal segmental glomerulosclerosis (FSGS), and diabetic nephropathy (DN) [3,4,5,6,7]. There are also convincing reports suggesting that the endothelin system might contribute to ischemia–reperfusion injury, acute graft rejection and deterioration of graft function [8,9,10,11]. This work aims to shed some light on both physiology of the ETAR/ETBR pathway and the effects of ET system inhibition. We collected the achievements in this field involving mainly ongoing and completed clinical trials that focus on using ET system inhibitiors in kidney diseases.

## 2. Role of Endothelin Receptors Pathway, and Their Distribution

### 2.1. Endothelin Pathway in Vascular System

**Figure 1:**
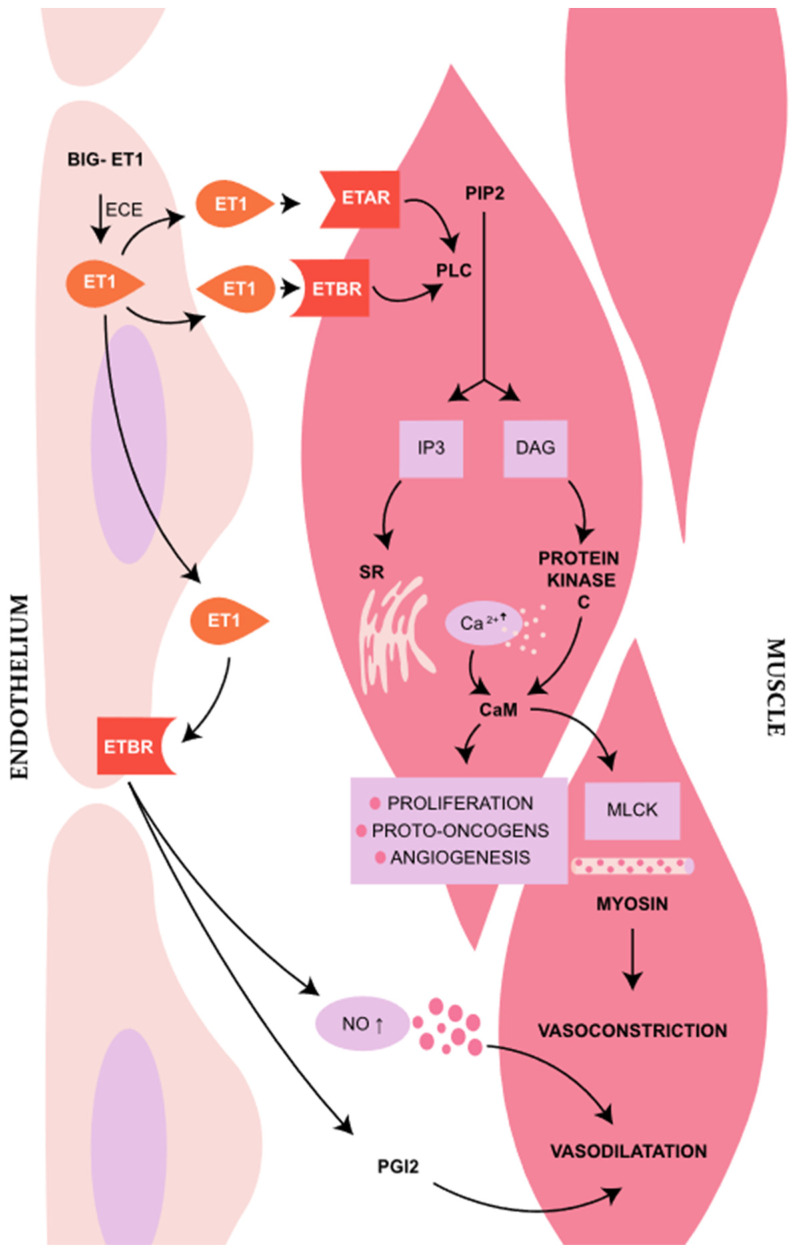
The scheme presents vascular endothelin pathway. MLCK—myosin light-chain kinase, CaM—calmodulin, PIP2—phosphatidylinositol 4,5-bisphosphate, IP3—inositol trisphosphate, DAG—diacylglycerol, SR—sarcoplasmatic reticulum, ECE—endothelin converting enzyme, PLC—phospholipase C, PGI2—prostaglandin I2. The illustration was made in Adobe Illustrator Program.

The ETs precursor (big-ET1) is synthesized in endothelin cells and subsequently converted into active endothelin 1 (ET-1) by endothelins’ converting enzymes (ECE). Activated ET1 binds to two seven transmembrane G-protein-coupled receptors—ETAR and ETBR. The signaling via receptors leads to the activation of phospholipase C (PLC), transforming the molecule of phosphatidylinositol 4,5-bisphosphate (PIP2) into inositol trisphosphate (IP3) and diacylglycerol (DAG). IP3 activates its receptor, located on the sarco-/endoplasmic reticulum by releasing Ca^2+^ into the cytosol. Activation of ETAR leads to an increase in intracellular calcium level inducing vasoconstriction. However, the activation of ETBR causes a release of nitric oxide (NO) and prostacycline 2, activating vasodilatation [2,12,13] (Figure 1).

### 2.2. Endothelin Pathway in Glomeruli

**Figure 2:**
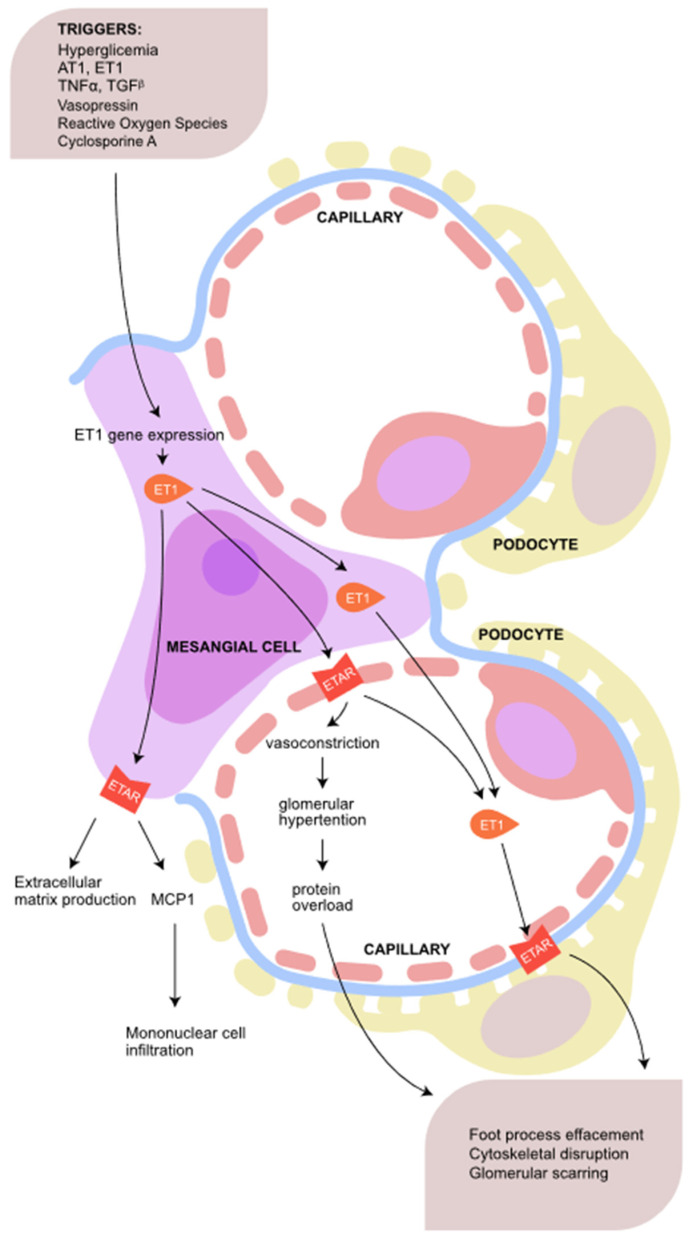
The scheme presents the endothelin system pathway in glomeruli. MCP1—monocyte chemoattractant protein-1. The illustration was made in Adobe Illustrator Program.

ET-1 is produced on both sides of the GBM—by endothelium cells and podocytes, affecting ETAR and ETBR by the autocrine and paracrine pathway. Activation of ETAR promotes mitogen-activated protein kinases (MAPKs) p38 and p44/p42 pathway, which regulate proliferation, cell survival, gene expression and apoptosis. Moreover, ETAR activation induces inflammation by releasing NF-kappaB, and causes disruption of the F-actin cytoskeleton and slit diaphragm dysfunction by releasing rho kinase and PI3 kinase. However, mesangial cells significantly may contribute to ET-1 production in response to various triggers. ET-1 stimulates mesangial cell proliferation and secretion of monocyte chemoattractant protein-1 (MCP-1), leading to an infiltration of inflammatory cells. Moreover, ETAR activation leads to the vasoconstriction of efferent arteriole, glomerular hypertension, and protein overload. Consequently, the process leads to glomerular injury and sclerosis (Figure 2) [12].

### 2.3. Endothelin Pathway in Renal Medullary

**Figure 3:**
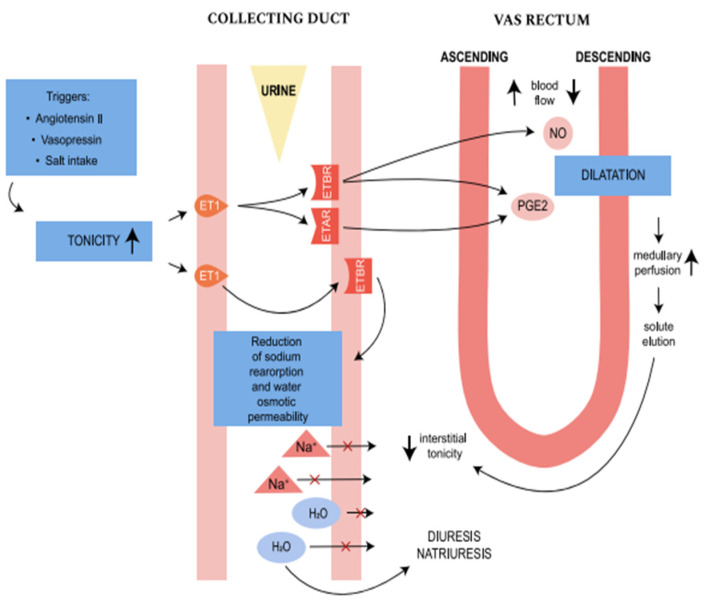
The scheme presents the endothelin system pathway in renal medullary. The illustration was made in Adobe Illustrator Program. The arrows with “×” means blockade of water and sodium reabsorption.

In the renal medulla, ET-1 release is stimulated by an increase in medullary interstitial tonicity due to high salt intake, vasopressin, or high concentration of AT1. ET-1 is produced mostly in the inner medullary collecting ducts. It binds with both ETBR and ETAR, inducing the production of NO and prostaglandin E2 (PGE2), causing vasodilatatory effects. NO and PGE2 increase medullary perfusion and induce solute washout in descending vasa recta, contributing to a decline in tonicity and eventually increasing diuresis and natriuresis. Activation of ETBR in collecting duct endothelium inhibits sodium reabsorption and reduces water osmotic permeability which also contributes to intensifying diuresis and natriuresis (Figure 3) [14].

### 2.4. ETAR and ETBR Distribution

The experimental study conducted on rats characterized the distribution of ETAR and ETBR in the vascular and tubular system. Wendel et al. used specific antibodies against ETAR and ETBR and the Western blot method. They reported that in the renal cortex, both ETARs and ETBRs were mainly located in the smooth muscles of the vascular system. In the interlobar and arcuate arteries only ETARs were expressed. ETARs evident expression was observed on mesangial cells and pericytes of descending vasa recta. In the renal medulla the expression of ETAR was located in distal tubules and cortical collecting ducts, while expression of ETBR was observed mainly in the epithelial cells of the proximal tubules and inner medullary collecting ducts [15]. The study suggests that both ETAR and ETBR regulate the renal cortical vasoconstriction, but in the renal medulla the effect of both receptors is differential (Figure 3). Activation of ETAR seems to stimulate the pathologic process, while ETBR has a protective role through vasodilatory and antifibrotic effects by initiating NO synthesis [1,12,15].

Drakopoulos et al. studied the expression of ETAR and ETBR in 37 human biopsies with different types of glomerulonephritis (GN) and proteinuria, together with urinary ET-1 concentration. ETAR and ETBR were analyzed by immunohistochemistry in the kidney sections and ET-1 urine concentration was measured in 24 h urine samples with specific radioimmunoassay. The results were evaluated concerning the degree of proteinuria. The expression of ETAR and ETBR were significantly higher in patients with GN compared to the controls. ET-1 concentration in the urine was positively correlated with the degree of proteinuria. In addition, ETBR expression was higher in patients with nephrotic syndrome and decreased during remission [16]. Dhaun et al. assessed plasma and urine endothelin-like domain peptide (ELDP) and C-terminal prot-ET-1 in CKD patients. The increased concentration correlated with higher CKD stage and proteinuria. The study suggested that the activation of the ET system contributes to the development and progression of CKD and proteinuria. Then, in a randomized, double-blind crossover study, they assessed the effects of a 6 week treatment with either a placebo, sitaxentan (ETAR antagonist), or nifedipine. Their research concluded that ELDP and C-terminal prot-ET-1 could be potential biomarkers of CKD and evaluate responses to the therapy with ETAR antagonists [17].

### 2.5. Role of ETAR and ETBR Activation

ET-1 as a potent vasoconstrictor and mitogen is involved in the control of systemic and microcirculation. ETAR and ETBR pathways regulate vascular tone. Activation of ETAR localized in efferent arteriole leads to constriction, glomerular hypertension, and protein traffic. ETBR has an opposite, protective role by releasing NO and prostacyclin. Selective ETAR blockade decreases systemic and glomerular hypertension by dilatation of efferent arteriole and vasa recta, having a protective effect so long as hypotension is prevented. Concomitant ETBR blockade is not desirable, because it may intensify vasoconstriction and retention of fluid and sodium. In regards to the non-hemodynamic effect of ETAR-blockade, it decreases the proliferation of mesangium, extracellular matrix production, and mutes’ inflammation. Due to the reduction in proteinuria, it protects podocytes from its consequences, such as foot process effacement and scarring. ETAR is also involved in collagen deposition processes, apoptosis, and fibrosis of interstitium; therefore, its inhibition has a positive and multidirectional effect [2,12,13,14].

## 3. Potential Protective Role of ETAR Inhibition in Chronic Kidney Disease

### 3.1. Analysis of ETAR Inhibition in Nephropathy IgA

IgAN is the most prevalent primary GN worldwide. In Europe, it comprises 4–25% of all GNs, in Japan, 25–60%, and less frequently in Africa. It is one of the most frequent GNs progressing to end-stage renal disease (ESRD) with a ten-year risk of ESRD varying between 5% and 60% [18]. Abnormal galactose-deficient IgA1 (Gd-IgA1) plays a key role in the pathogenesis, initiating immunological complexes with auto-antibodies IgG and IgA. The complexes are deposited in the glomerular mesangium, subsequently activating complement, local cytokines, the proliferation of mesangial cells, and the loss of podocytes [18,19]. ET-1 is elevated in monocytes, neutrophils, and the kidneys in patients with IgAN [20,21]. Furthermore, renal ET-1 concentration measured in biopsies with IgAN correlates with albuminuria and one year risk of progression [21,22,23]. Clinical data have shown that the blockade of both endothelin and angiotensin II type receptors (AT1R) protect podocytes, reduces mesangial proliferation and prevents glomerulosclerosis. Sparsentan is a dual endothelin and angiotensin receptor antagonist selectively targeting ETAR and AT1R. Numerous clinical trials are focusing on sparsentan as a first-line protective treatment reducing proteinuria in GN [5,6,18,24]. Sparsentan received accelerated approval by the FDA in kidney disease protective treatment, in February 2023, for reducing proteinuria in IgAN [18,25]. In an international, randomized, double-blind, active-controlled study PROTECT examined 380 participants randomly assigned to sparsentan 400 mg versus irbesartan 300 mg in adults with biopsy-proved IgAN and proteinuria of 1.0 g/day or higher. Patients were stratified by glomerular filtration rate (GFR 30 to <60 mL/min per 1.73 m^2^ and ≥60 mL/min per 1.73 m^2^) and urinary protein-to-creatinine ratio (UPCR ≤ 1.75 g/day and >1.75 g/day). The decrease in UPCR from baseline was significantly higher in the group receiving sparsentan, resulting in a relative reduction of 41% after 36 weeks of treatment. The final endpoint analysis of long-term nephroprotective action of sparsentan is set after two years of treatment. There were no severe adverse events resulting in treatment discontinuation. The most common adverse reactions of sparsentan included peripheral edema, hypotension, dizziness, hyperkalemia, and anemia as an effect of hemodilution [5]. The trial was extended to the OLE Sub study modified by co-administration of dapagliflozin and sparsentan (ClinicalTrials.gov ID NCT03762850). The results have not been published so far. SPARTACUS is another ongoing clinical trial evaluating the safety of the co-administration of sparsentan with dapagliflozin (ClinicalTrials.gov ID NCT05856760). SPARTAN examines sparsentan as a first-line treatment in IgAN in patients not receiving any treatment with renin-angiotensin-aldosterone (RAA) blockers or sodium-glucose co-transporter 2 (SGLT-2) inhibitors (ClinicalTrials.gov ID NCT04663204 Table 1).

### 3.2. Analysis of ETAR Inhibition in Focal Segmental Glomerulosclerosis

FSGS is a leading cause of nephrotic syndrome in adults with a substantial risk of progression to ESRD [4]. FSGS can be classified as primary, secondary, or genetic. Pathological changes in FSGS include podocyte injury, mesangial matrix expansion, capillary occlusion, and tuft adhesion. Several reports suggest that the upregulation of the ET pathway plays a significant role in FSGS. ET-1 is increased in aging-associated FSGS, enhances podocyte damage, and promotes glomerulosclerosis which might be correlated with upregulation of p21 in podocytes [26,27,28,29]. Furthermore, ETAR blockade partially prevented glomerulosclerosis development and decreased proteinuria in aging-associated FSGS [28,29]. DUET is a randomized, double-blind, active-control study that compares the safety and efficacy of sparsentan with escalated doses (200, 400 or 800 mg/d) versus irbesartan (300 mg/d) in 109 participants with biopsy proven FSGS, an eGFR > 30 mL/min/1.73 m^2^ and UPCR ≥ 1 g/d. The results present a significantly greater reduction in UPCR and more cases of partial remission (28% vs. 9%) in the sparsentan treated group at eight week of therapy. Within nine months of therapy, 52.8% of patients achieved partial remission which was associated with the prevention of eGFR decline. The most common adverse effects were headache, peripheral edema, upper respiratory infections, hyperkalemia, and hypotension [6,7]. Another trial DUPLEX is a multicenter, international, phase three randomized, double-blind, active-controlled study comparing sparsentan to irbesartan in FSGS. The study enrolled 371 patients with biopsy-confirmed FSGS or genetic mutation in a podocyte protein associated with FSGS, UPCR ≥ 1.5 g/g, and eGFR ≥ 30 mL/min/1.73 m^2^. At the final analysis, after 108 weeks of treatment, the reduction in proteinuria was greater in the sparsentan-treated group; however, there was no significant differences in eGFR decline between these two groups [24,30] (Table 1).

### 3.3. Analysis of ETAR Inhibitionin Diabetic Nephropathy

Several experimental studies have reported a beneficial influence of ETAR-inhibition therapy in diabetic nephropathy (DN). Hyperglycemia promotes the production of ET-1 and contributes to the disruption of the actin cytoskeleton, apoptosis, and podocyte depletion [6]. Diabetic patients have increased immunostaining for ET-1 and ETAR in renal endothelial cells. Concentrations of ET-1 in plasma were correlated with urinary albumin excretion and a reduction in GFR in DN patients [31,32]. In a randomized, placebo-controlled, double-blind experimental study avosentan, a selective ETAR antagonist, added to standard angiotensin-converting enzyme inhibitor (ACEI) therapies significantly reduced albuminuria compared to placebo after 12 weeks of treatment in DN type I and II [33]. The ASCEND trial was a multinational, double-blind, placebo-controlled trial conducted on 1392 participants with type 2 diabetes. The patients randomly were administered avosentan 25 mg or 50 mg or placebo in addition to ACEIs. In avosentan-treated patients, the median reduction in UACR was significantly higher compared to placebo-treated patients (49.3% vs. 9.7%). The trial was terminated prematurely after four months due to numerous cardiovascular events, mainly fluid overload and congestive heart failure, which was observed in avosentan-treated patients [34]. Hoekman et al. decided to identify risk factors of heart failure after treatment with avosentan. The analysis reported that avosentan increased the risk of progression of congestive heart failure. The risk was higher in patients with lower baseline cholesterol levels, concomitant statin use and higher GFR. The mechanism of fluid retention during ETAR antagonist treatment is not well-known. It might be associated with low selectivity of avosentan and blockage of ETBR which has natriuretic and diuretic effects through the inhibition of sodium and water reabsorption [35]. Atrasentan is another an experimental selective ETAR inhibitor being studied in DN. The results from clinical trials presented that atrasentan reduces albuminuria, improves blood pressure control and lipid profiles, although adverse outcomes were fluid overload, edema, and reduction in hemoglobin concentration [3,36]. In the analysis of fluid retention predictors: higher atrasentan doses, lower eGFR, higher glycated hemoglobin, higher systolic blood pressure, and lower homeostatic metabolic assessment product were observed as risk factors. A higher dosage of atrasentan and decreased eGFR were also associated with anemia [37]. SONAR is a double-blind, randomized, placebo-controlled trial conducted from 2013 to 2017 in 689 centers at 41 countries (ClinicalTrials.gov Identifier: NCT01858532). The 2648 adult patients were randomly assigned to atrasentan (0.75 mg) or placebo. Participants were diagnosed with diabetes type 2 with an eGFR 25–75 mL/min per 1.73 m^2^ and UACR of 0.3–5 g/g. They firstly received the maximum tolerated doses of an RAA inhibitor for at least four weeks. Before random group assignment responders were given 0.75 mg of atrasentan daily. Those with a UACR decrease of at least 30% and no significant fluid retention were included into double-blind treatment. The primary endpoint was a composite of doubling of serum creatinine or ESRD. In the atrasentan group 79 responders (6%) achieved a primary renal endpoint event versus 105 of responders (7.9%) in the placebo group (*p* = 0.0047). Fluid retention and anemia were more frequent in the atrasentan group [4]. Zibotentan is an ETAR antagonist with no affinity for ETBR, studied in clinics for prostate cancer and heart failure but failed phase III trials. An experimental study on animal models combining the treatment of dapagliflozin and zibotentan was assessed. In the zibotentan group, bodyweight was numerically increased, but combining zibotentan with dapagliflozin for seven days prevented a change in hematocrit and weight gain. SGLT2 inhibitors produce an osmotic diuresis, and as a hypothesis, it might mitigate the fluid retention caused by ETAR antagonists [26]. In the ZENITH-CKD trial, zibotentan was assessed as a treatment of CKD as monotherapy and in addition to SGLT2 inhbitor—dapagliflozin (Clinical.Trials.gov NCT04724837). A total of 495 responders were randomly assigned to three groups: zibotentan 1.5 mg or 1.5 mg + dapagliflozin 10 mg and placebo + dapagliflozin 10 mg daily. The study concluded that the co-administration of SGLT2 inhibitor minimizes fluid retention and a drop of hematocrit [26,38]. In an experimental study on mice triple therapy consisting of empagliflozin, atrasentan and ramipril achieved the greatest protective effect, but the adverse events of fluid retention were not assessed [38] (Table 1).

### 3.4. ETAR Inhibitionin Other Nondiabetic Nephropathy

The results from the phase II, single-center, randomized, placebo-controlled, double-blind trial ZEBRA1 suggest that zibotentan improved eGFR in CKD secondary to systemic sclerosis, compared to placebo. However, only 13 patients were recruited for ZEBRA1, so research needs to continue [39]. Furthermore, sparsentan is being examined in ANCA-associated vasculitis (AAV). The currently published results report that patients with AAV presented two-fold higher plasma ET-1 concentrantions compared to healthy volunteers, which was associated with greater vascular stiffness and reduced endothelial function. The study demonstrates that ETAR blockade approaches reduced pulse wave velocity and increased tissue polypeptide antygen in AAV patients. The researchers conducted a randomized, double-blind active controlled study in patients with long-term remission. Before and after six weeks of treatment with an ETAR-antagonist versus irbesartan forearm blood flow will be assessed. This will assess the endothelial function improvement after treatment [40,41]. Alport syndrome (AS) is a hereditary disease caused by mutations in type IV collagen genes leading to defective GBM. There is no preventive or curative treatment for AS. New treatment strategies are still under investigation. Experimental research on AS mice compared the effect of sparsentan and losartan on lifespan and proteinuria. Sparsentan in a dose of 120 mg extended lifespan, attenuated development of proteinuria, and reduced strial capillary basement membrane width. Moreover, sparsentan significantly delayed noise-induced hearing loss, in comparison to losartan which had no such effect [42]. Already, there is an ongoing clinical trial EPPIK in the pediatric population and AFFINITY in adults which recruits also patients with AS, currently the results have not been published [43] (Table 1).

## 4. The Role of the Endothelin System after Kidney Transplantation

Endothelin levels increase after graft transplantation and may play a significant role in the development of post-transplant complications; ETAR expression as induced by inflammation and ischemia–reperfusion injuries, which increases targets for auto-antibodies. Experimental trials have reported that calcineurine inhibitors treatment promotes the expression of ET-1 by endothelial cells and proximal tubule cells, which might be associated with higher serum creatinine concentrations, renal fibrosis, and impairment of renal function [44,45,46,47,48]. An analysis of ETAR in allograft biopsies of 154 patients showed that the location of ETAR in renal particular specimens might be associated with a higher risk of progression to graft failure. Patients with positive staining of ETAR in small and intermediate arteries developed acute tubular necrosis and antibody mediated rejection [9]. Antibody-mediated rejection is considered to be the main pathway leading to graft deterioration or loss after kidney transplantation. In another investigation, almost half of the kidney recipients were anti-ETAR positive before the transplantation which was associated with worse graft function at the one-year follow-up compared to the anti-ETAR negative group [11]. Also, expression of ETAR in glomeruli and renal blood vesseles may be related to damage in the course of antibody-mediated rejection and acute tubular necrosis [9,10,11]. It has been reported that ETAR inhibition treatments in lung transplantation improve pulmonary function after ischemia–reperfusion injury [49]. In cardiac transplantation, vascular ET-1 expression was also associated with acute rejection, and interstitial ET-1 was connected with ischemic trauma and vasculopathy development [50]. Additionally, in the research conducted on rats after liver transplantation, ETAR inhibition improved liver graft morphology and function, possibly through influence on microcirculatory lesions [51]. Knowledge of ETAR expression and its inhibition may play a notable role in the prevention of post-transplant complication. However, further research is required in this field.

## 5. Conclusions and Future Directions

Endothelin system inhibition therapy is a promising direction in protective treatment preserving renal function in CKD. A growing body of evidence suggests that selective ETAR antagonists play a significant role in reducing proteinuria, preserving renal function, and delaying the progression of CKD, with even greater effectiveness to RAA inhibition. There are numerous ongoing and completed clinical trials on ETAR antagonists, reporting positive results, in IgAN, FSGS, DN, AS, sclerodermia and AAV nephropathy. However, in patients with congestive chronic heart failure and CKD the treatment should be provided with the highest caution. The most frequent side effect is gaining weight due to fluid overload, which may be especially significant in patients with lower eGFR. This is why diuretic co-administration should be considered. There are also some questions about whether selective ETAR antagonists should be co-administered with SGLT2 inhibitors or an ACEI/ARB. RAA inhibitors are still the first line treatment in reducing proteinuria, but selective ETAR antagonists might bring benefits for patients with intolerability to ACEI/ARB or tendency for hyperkalema. Some ETAR antagonists (for example sparsentan) have dual action for AT1R and ETAR. The molecule achieved positive effects in SONAR, DUET, and DUPLEX trials. There are also some reports showing that endothelin system may play a significant role in post transplant graft complications, associated with acute rejection. It is probable that ETAR inhibition might have a positive effect in preserving renal graft function and the prolonging of graft survival. The endothelin system seems to play a noticeable role in ischemia–reperfusion injuries and acute rejecton which was also described in cardiac, pulmonary, and liver transplantation. However, there is still a lack of research on ETAR inhibitors on long-term graft function, and this field needs to be further examined.

## Figures and Tables

**Table 1:** Endothelin inhibitors in kidney diseases (based on ClinicalTrials.gov).

Clinical Trial.gov Identifier	Intervention	Description	Additional Information	Measured Outcome	Enrollment
Nephropathy IgA					
ALIGN NCT04573478 (active, not recruiting)	Atrasentan in patients with IgAN (0.75 mg of atrasentan or placebo for 132 weeks)	A phase 3, double-blind, placebo-controlled study to determine the efficacy and safety profile of atrasentan comparing to placebo in IgAN at risk of progressive loss of renal function.	Patients receive maximal doses of RAA inhibition, additional subjects receive stable dose of SGLT2— inhibitors	Change in UPCR and eGFR, incidence of treatment-emergent adverse events	320
ASSIST NCT05834738 (recruiting)	Atrasentan in patients with IgAN Patient will be randomized to sequence AB or BA: A:12 weeks—0.75 mg atrasentan B: 24 weeks—placebo or B: 12 weeks—placebo A: 24 weeks—0.75 mg atrasentan	A phase 2, double-blind, placebo-controlled crossover study to evaluate safety and efficacy profile of atrasentan in patients on therapy of maximum RAA blockade and SGLT2 inhibitors	Patients should undergo at least 8 weeks therapy of SGLT2—inhibitors and UPCR > 0.5 g per day, UPCR > 0.85 g per day at screening and eGFR 30 mL/min/1.73 m^2^ or more	Change in UPCR and incidence of treatment-emergent adverse events	52
SPARTACUS NCT05856760 (active, not recruiting)	Sparsentan 400 mg in patients with IgAN	A phase 2, multicentered, single-group study to assess safety and effect profile of sparsentan in patients at high risk of progression	Inclusion criteria: stable doses of RAA inhibitor and SLGT2 inhibitor for at least 12 weeks, eGFR 25 mL/min/m^2^ and more, UACR at least 0.3 g/g	Change in urine albumin-creatinine ratio (UACR), change in eGFR, change in blood pressure from baseline at week 24	60
PROTECT NCT03762850 (active, not recruiting)	400 mg of sparsentan vs. 300 mg of irbesartan OLE Sub study: dapagliflozin 5 mg + sparsentan 400 mg vs. sparsentan 400 mg for 12 weeks	A randomized, phase 3, multicenter, double-blind, parallel-group, active-control study of the efficacy and safety of sparsentan in IgAN	Inclusion criteria: proteinuria at least 1 g per day, eGFR at least 30 mL/min/m^2^, on stable dose of ACEI/ARB for 12 weeks prior to screening	Change in UPCR at week 36, change in eGFR at week 110	406
IgA Nephropathy, focal segmental glomerulosclerosis, Alport Syndrome, Diabetic Kidney Disease					
AFFINITY NCT04573920 (active, not recruiting)	Atrasentan 0.75 mg for 52 weeks, in patients with FSGS and well tolerated treatment the dose will be titrated up to 1.5 mg per day	A phase 2, open-label, non-randomized to determine safety and efficacy profile of proteinuric GN	Inclusion criteria: eGFR at least 30 mL/min/m^2^ IgAN UPCR 0.5–1 g/g, FSGS more than 1 g/g, AS > 0.5 g/g, for diabetic kidney disease UACR at least 0.5 g/g, eGFR at least 45 mL/min/m^2^ and stable dose of SGLT2-inhibitor	Change in UPCR at week 12 (IgAN, FSGS), change in albuminuria at week 12 (diabetic kidney disease), change in proteinuria for FSGS patients at 1.5 mg dose to up to week 24 and 30	80
Focal segmental glomerulosclerosis, minimal change disease (MCD), IgA Nephropathy, IgA-associated Vasculitis, Alport Syndrome					
EPPIK NCT05003986 (recruiting)	Sparsentan 800 mg (in FSGS and minimal change disease); sparsentan 400 mg (IgAN, AS, IgA—vasculitis)	A phase 2, open-label, 112-week, single-arm, cohort study to evaluate safety, efficacy, and pharmacokinetics of sparsentan in pediatric patients	Pediatric population at age 1–18, biopsy proven FSGS, MCD, AS, IgA—vasculitis, IgAN or genetic testing for pathogenic X-linked collagen/genetic mutation in podocyte protein associated with FSGS or MCD	Change in UPCR at 108 weeks, incidence of treatment-emergent adverse events leading to discontinuation of a treatment	67
Focal Segmental glomerulosclerosis					
DUET NCT01613118 (completed)	Sparsentan 200 mg; 400 mg; 800 mg versus irbesartan 300 mg	A phase 2, randomized, double-blind, active-control, dose-escalation study to evaluate sparsentan in FSGS	In eight week study sparsentan reduced UPCR better than irbesartan, in DUET open-label extension over >4 years reduction in proteinuria was sustained and no new unexpected adverse events were observed	Change in UPCR in patients with FSGS after 6 weeks of treatment	109
DUPLEX NCT03493685 (active, not recruiting)	Sparsentan 800 mg vs. irbesartan 300 mg	A phase 3 randomized, multicenter, double-blind, parallel, active-control study of the effect of sparsentan in FSGS	After 36 weeks of therapy 42% of patients achieved partial remission of proteinuria versus 26% of irbesartan treated patients (*p* = 0.0094)	Decrease in eGFR over 2 years, percentage of patients achieving partial remission (UPCR ≤ 1.5/g and reduction > 40% from baseline	371
ANCA—associated vasculitis					
SPARVASC NCT05630612 (active, not recruiting)	Sparsentan or irbesartan for 6 weeks	A phase 2, randomized, double-blind, active control, parallel group study assessing ETAR and AT1R antagonism on endothelial function and vascular stiffness	Patients with ANCA-associated vasculitis and longtime remission; forearm blood flow in response to acetylcholine and sodium nitroprusside is going to be measured; patients will receive bradykinin to measure fibrinolytic capacity. Subjects will have 24 h measurement of blood pressure, arterial stiffness and proteinuria	Change in acetylcholine-mediated forearm blood flow vasodilatation, secondary outcome—fibrinolytic capacity, blood pressure, arterial stiffness, systemic hemodynamics, proteinuria	32
Diabetic nephropathy					
RADAR NCT01356849 (completed)	Atrasentan 1.25 mg/0.75 mg vs. placebo for 12 weeks	A prospective, phase 2, randomized, double-blind, placebo-controlled multicenter study to determine efficacy and safety of atrasentan in reducing residual albuminuria in diabetic type 2 nephropathy	Patients with diabetic nephropathy who are treated with maximum tolerated RAA inhibitor	Change to week 12 in UACR	149
SONAR NCT01858532 (terminated)	Atrasentan 0.75 mg vs. placebo for up to 52 months	A prospective, phase 3, randomized, double-blind, placebo-controlled, multicenter in patients with type 2 diabetic nephropathy	Atrasentan reduced the risk of renal events; adverse effects were fluid retention and anemia were more frequent with atrasentan group comparing to placebo	Time to occurrence of the composite of renal endpoint—doubling serum creatinine, eGFR < 15 mL/min/m^2^, dialysis, renal transplantation or renal death	2648
Chronic kidney disease					
ZENITH-CKD NCT04724837 (completed)	(1) Zibotentan 0.25 mg and dapagliflozin 10 mg (2) zibotentan 1.5 mg and dapagliflozin 10 mg (3) Placebo and dapagliflozin 10 mg	A phase 2b, multicenter, randomized, double-blind, active-control, parallel group, dose-ranging study to assess efficacy and safety of zibotentan and dapagliflozin in patients with CKD and eGFR ≥ 20 mL/min/m^2^	Inclusion criteria: eGFR ≥ 20 mL/min/m^2^, urinary albumin-to creatinine ratio from 150 mg/g to 5000 mg/g inclusive, no prior treatment of SGLT-2 inhibitors	Change in urinary albumin to creatinine ratio to week 12; change in blood pressure, eGFR, incidence of adverse events	447
Scleroderma-associated nephropathy					
ZEBRA NCT02047708 (completed)	Zibotentan vs. placebo	A phase II, single center, randomized, placebo-controlled, 3-part trial to assess efficacy and safety profile of zibotentan in patient with renal disease secondary to scleroderma	ZEBRA 1 for patient with mild or moderate kidney disease ZEBRA 2A for patients with scleroderma renal crisis but not requiring dialysis ZEBRA 2B for patients with renal crisis and requiring dialysis	sVCAM 1 soluble vascular cell adhesion molecule as a biomarker of renal involvement in scleroderma in 12 weeks	27

## Data Availability

Not applicable.

## Abbreviations

AAV	ANCA-associated vasculitis
ACEI	angiotensin-converting-enzyme inhibitors
ANCA	anti-neutrophil cytoplasmatic antibodies
ARB	angiotensin receptor blockers
AS	Alport syndrome
CaM	calmodulin
CKD	chronic kidney disease
DAG	diacylglycerol
DN	diabetic nephropathy
ECE	endothelin converting enzyme
eGFR	estimated glomerular filtration rate
ESRD	end-stage renal disease
ET	endothelins
ET-1	endothelin 1
ETAR	endothelin A receptor
ETBR	endothelin B receptor
FSG	focal segmental glomerulosclerosis
GBM	glomerular basement membrane
Gd-IgA1	galactose-deficient immunoglobulin A1
GN	glomerulonephritis
IgAN	immunoglobulin A nephropathy
IP3	inositol trisphosphate
MAPK	mitogen-activated protein kinases
MLCK	myosin light-chain kinase
NO	nitric oxide
PGE2	prostaglandin E2
PIP2	phosphatidylinositol 4,5-bisphosphate
RAA	renin–angiotensin–aldosterone
SGLT2i	sodium-glucose co-transporter 2 inhibitor
SR	sarcoplasmatic reticulum
UACR	albumin-to-creatinine ratio
UPCR	urinary protein-to-creatinine ratio
